# An educational program of reducing moral distress (PRMD) in nurses; designing and evaluating

**DOI:** 10.1186/s12909-023-04445-4

**Published:** 2023-07-11

**Authors:** Nahid Tavakol, Zahra Molazem, Mahnaz Rakhshan, Omid Asemani

**Affiliations:** 1 Shiraz University of Medical Sciences, Shiraz, Iran; 2 Present Address: Medical Ethics Research Center, Jahrom University of Medical Sciences, Jahrom, Iran; 3 Department of Nursing, School of Nursing and Midwifery, Shiraz University of Medical Sciences, Shiraz, Iran; 4 Community Based Psychiatric Care Research Center, Shiraz University of Medical Sciences, Shiraz, Iran; 5 Department of Medical Ethics and Philosophy of Health, Faculty of Medicine, Shiraz University of Medical Sciences, Shiraz, IR Iran; 6 Center for Interdisciplinary Research in Islamic Education and Health Sciences, Shiraz University of Medical Sciences, Shiraz, IR Iran

**Keywords:** Morals, Ethics, Nursing, Qualitative research, Education, Program development

## Abstract

**Background:**

Moral distress is common phenomenon that has negative consequences on nurses, patients, and healthcare systems. This study aims to design and evaluate an educational program to reduce moral distress in nurses.

**Methods:**

This multiphase mixed-method study was done in three stage on February 2021 in Shiraz/Iran. In pre-implementation stage, a content analysis study was conducted on 12 participants were interviewed using purposive sampling and then the program was designed according to qualitative data, panel of expertise and literature review according the seven steps of Ewles and Sminett’s model and implemented in one group on 40 nurses using a quasi-experimental design. In Post-Implementation stage, effectiveness of program was evaluated through quantitative and qualitative methods. Quantitative data were gathered by Hamric's 21-question moral distress questionnaire analyzed via SPSS v.25 and analysis of variance repeated measures test. Also, a content analysis study was conducted on 6 PRMD participants using purposive sampling. In Program evaluation stage, convergence of quantitative and qualitative data and the effects of the program were examined. Trustworthiness of qualitative data was accomplished by Lincoln and Guba criteria.

**Results:**

First quantitative study revealed the causes of moral distress consisted of deficiency in professional competency, unsuitable organizational culture, personal factors, environmental and organizational factors, management factors, insufficiencies in proficient and efficient communication and nurses' observation of moral dilemma. Results of quantitative stage showed that there was a significant difference (*p* < 0.05) between the mean score of moral distress before, after, 1 and 2 months after the intervention. The participants in secondary qualitative stage, reported increasing their moral knowledge and skills, improving ethical climate, and moral empowerment.

**Conclusion:**

The use of different educational tools and teaching methods and the participation of managers in designing strategies had a very effective role in the effectiveness of this educational program**.**

## Introduction

Moral distress is a common phenomenon in nurses that has negative consequences on nurses, patients, and healthcare systems. This phenomenon occurs when the nurse recognizes the right moral action, but cannot act on it due to organizational and professional limitations [1, 2]. Studies have reported its prevalence in nurses of all wards, including critical wards [3], medical-surgical wards [4], oncology [5], pediatrics [6] and psychiatry [7].

Various factors such as inadequate treatment by nurses [8], negligence and medical errors [9], lack of time, the unethical performance of other people [10], lack of continuity of care, and poor communication between physicians and nurses [11], medication errors [12] Lack of knowledge, lack of competence and professional skills, lack of human resources and high workload [13, 14] play a role in creating moral distress.

Moral distress as a fundamental problem not only threatens the job satisfaction of nurses, but also affects the ability to perform daily activities and responsibilities of nurses and causes issues such as increasing care errors, weakness feeling, and lack of authority [15].

Few interventions and models have been designed to manage and reduce moral distress, including the "4A" model and the "CERN" model (Clinical Ethical Residency for Nurses). "Model 4A" was introduced in 2004 by the American Association of Critical Care Nurses (American Association of Critical Care Nurses) to reduce moral distress, which is a framework for nurses in critical wards to identify and reduce moral distress [16]. Also, the CERN training model in America was designed to reduce the moral distress of nurses in the acute care department. This model is an educational curriculum designed to reduce moral distress.

These two models are not designed based on the context so the designers of the CERN model suggested that to design ideal moral programs in healthcare systems, a detailed analysis of the existing situation should be done and the knowledge of existing ethical thinking, clinical knowledge, organizational environment and available resources should be investigated [17].

Despite the importance and the implications of moral distress for the quality of healthcare [18], the difficulties affirmed by nurses when they are faced with ethical issues and dilemmas or they are called upon to make ethical decision [14] this phenomenon is still a partly unexplored field of research because few studies have designed interventional program for decreasing moral distress. Moreover, at a conceptual and theoretical level, there remains a knowledge gap that prevents action from being taken on moral distress at the educational, political, organizational, and practical levels.

According to a study conducted by Mohammadi et al., the level of moral distress among Iranian nurses was found to be moderate to high [19] and also other studies confirm this finding [20, 21]. Problems such as vagueness of role definition, high workload, a severe shortage of nurses [22], non-participation in policies and decisions, an authoritarian hierarchical system in the relationship between doctors and nurses [23] and lack of ethics committees to conduct ethical consultations [24] for nurses in Iran, increase their moral distress, Furthermore unprecedented challenges to healthcare system in Iran due to COVID-19 pandemic, nurses have faced numerous ethical dilemmas and complex decision-making processes, leading to increased moral tension and moral distress [25].

Therefore, according to the conditions of Iranian nurses, who are prone and vulnerable to moral distress, conducting interventions based on the culture and values of nurses [26] and designing a program based on the Iranian-Islamic culture of nurses, the existing setting and facilities are necessary. Arrives For this purpose, the Multiphase mixed design method was chosen because it is most compatible with the purpose of the research.

This method is a complex design that is used in the evaluation of intervention programs. In this study, qualitative data is first collected and analyzed and a program is designed based on this stage, and then the designed program is implemented during a quantitative study, then the evaluation of the program is done during the qualitative and quantitative stage [27].

### Objective

The purpose of this study was to design a program to reduce moral distress in nurses according to the clinical setting of Iran and according to the cultural context and organizational structure of Iranian hospitals, and then to implement and evaluate the effectiveness of the designed program.

## Method

### Study design

We utilize a multiphase mixed-methods plan to reach the goals of the research (Table 1). Our mixed-methods plan, based on philosophical presumptions driven by pragmatism, incorporates the collection and integration of qualitative and quantitative information for complementarity to fortify interpretation and give a more total understanding of a set of interconnected research questions [28].

**Table 1:**
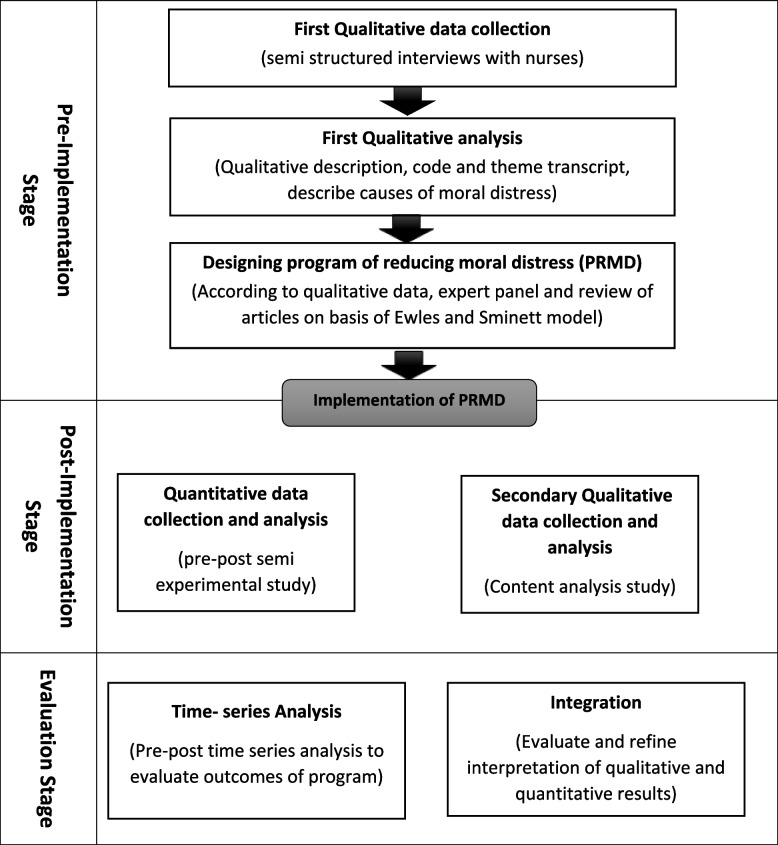
Mixed-method multiphase study design

We collect data over 3 stages. In the Pre-implementation stage, qualitative content analysis study. The program of reducing moral distress (PRMD) designed on basis of the qualitative finding according to the steps of Ewles and Sminett Model (2010) [29]. In the Post-implementation stage, a program was implemented in one group pre-post semi-experimental design on 40 nurses. Also, secondary qualitative study via semi-structured Interviews were done following the implementation of the intervention on 6 participants of program. In the program evaluation stage; we mixed both qualitative and quantitative post-implementation data to comprehensively evaluate and refine the intervention and to determine evidence-based implications for PRMD.

### Setting and participants

#### Pre-implementation stage

##### First qualitative phase

This phase has been conducted in 3 general large hospitals (Namazi, Faghihi and Ali-Asghar) and 1 psychiatric hospital (Ibn sina) affiliated to Shiraz University of medical sciences, Iran on February 2021.

The nurses were recruited based on inclusion criteria consist of (1) licensed as an academic nurse, (2) having at least 6 months of work experience, (3) not participating in problem-solving and ethical decision-making courses. Exclusion criteria included not working in the inpatient wards and with patients. Twelve nurses participated and were selected using purposive sampling. Maximum variation sampling was used to identify and expand the range of variation in moral distress experienced by nurses.

#### Post -implementation stage

##### Quantitative phase

Due to accurately measuring the effects of the program and implementing the centralized interventions, it was necessary to carry out the interventions in one center. Considering that one setting where there are many moral challenges and high levels of moral distress is psychiatric wards [30]; to better examine the effects of PRMD, the largest psychiatric center in the south of Iran was selected that have 223 bed with 121 psychiatric nurse. In this phase, a sample size of 40 achieves 81% power to detect a mean of paired difference of 7.0 with an estimated standard deviation of the difference of 15.0 and with a significance level (alpha) of 0.05 using a tow-sided paired T-Test.

These participants will be selected using a random sampling of volunteers to participate in the study.

##### Secondary qualitative phase

This phase has been conducted in 1 general large psychiatric hospitals (Ibn sina) affiliated to Shiraz University of medical sciences, Iran. The nurses were recruited based on inclusion criteria consist of (1) complete participation in all interventions of program (2) willingness to share one’s experiences with the researchers. The subjects who were not willing to be interviewed or continue their participation in the study were excluded. Six nurses participated and were selected using purposive sampling.

### Data collection and data analysis

#### Pre-implementation stage

##### First qualitative phase

In this phase, open-ended questions and the interview guide were used. Face-to-face semi-structured interviews were conducted and digitally audiotaped after coordinating the time of the interview with participants in a suitable and private place in the ward. The duration of the interviews was between 45 and 60 min Each interview lasted from 40 to 60 min and began with a general question “Can you describe your experiences of a work shift in which you faced or cared for patients?” Subsequently, the interviewer asked more specific questions: “What are your experiences about the ethical issues when you faced and cared for patients?”, “What factors causes’ moral distress when face and care for patients?” Moreover, follow-up questions were asked to obtain more details about the objective of the study. Immediately after the completion of the interview, it was implemented verbatim and analyzed. The interviews continued until saturation was reached so that no new code was obtained in the last two interviews. Interviews continued until data saturation and when no new code was created in the last two interviews. The research team and two qualitative research experts examined the codes to verify that the data were saturated. The data were organized using MAXQDA 2010 distributed by VERBI.

The process of data analysis was carried out according to the steps suggested by Granheim and Lundman, which include 1- implementing the interview immediately after its completion, 2- reading the text for its general understanding, 3- determining semantic units and primary codes, 4- Classification of similar primary codes in themes, 5- determining the content hidden in the data [31].

Trustworthiness of the study was accomplished across four domains as described by Lincoln and Guba [32]. Credibility or confidence in the findings was established through explicit probes to get participants’ responses with greater precision and reflexivity to eliminate the researcher’s bias. Transferability was maintained by heterogeneous sampling to see the difference in experiences of the participants. Dependability or auditability was established throughout the analytic process, detailing decision rules and justifications via memo and reflexivity. Finally, for conformability, a peer check was conducted by the research team.

##### Designing Program of Reducing Moral Distress (PMRD)

After clarification of causes of moral distress according to Qualitative data, the PMRD was designed based on Ewles and Sminett Model. This model is ideal as a guide in planning effective programs for health promotion, which helps to plan accurately, in-depth, and in detail; according to the needs of the setting to solve the desired problem [29]. The PMRD implemented during 3 month on winter 2021.

### Ewles and Sminett Model

#### Stage 1: Identifying needs and priorities

The problems and needs of nurses related to moral distress were determined during the qualitative phase of the research by interviewing the participants. Then, these needs were prioritized by experts. They were selected from all hospitals covered by Shiraz University of Medical Sciences and from matrons, supervisors, nursing managers, and nursing professors. 15 experts participated in the need’s assessment.

#### Stage 2) Setting aims and objectives

After determining the priorities based on the need’s assessment, the general aims of the program were determined.

#### Stage 3) Deciding the best way to achieve the aims

In this stage, interventions were designed based on a literature review on moral distress in Iran and the world, and identified priorities were determined by experts. Three medical ethics specialists, one nursing PhD student and one psychiatric nurse (MSN) collaborated in educational sessions and training classes. Different teaching techniques such as lectures, PowerPoint, group discussion, and educational animation clips were used to enhance learning. Moral group meetings and narration and storytelling methods such as discussing moral distress experiences to dealing with MD were employed. The PRMD were presented in Table 2.

**Table 2:** Program of Reducing Moral Distress (PRMD)

Needs and problems perceived by participants		Aims	Interventions
1	Heavy workload	management of workload to reduce moral distress in nurses	- Holding 2 intra-ward meetings of nurses with managers to identify and correct processes that have increased the workload of nurses
2	Improper apportion duties		- Holding 2 intra-ward meetings of nurses with managers to identify and correct appropriate apportion duties methods in the ward
3	Inappropriate interpersonal interactions with patients and colleagues	Improving interpersonal interactions to reduce moral distress in nurses	- Holding an in-person educational session on interpersonal communication skills (patients, companions, and colleagues)
			- Presentation of educational clip in a virtual group, about interpersonal communication skills
4	conflict with the patient		- Conducting question and answer sessions and presenting cases and examples about communication skills in the virtual group
			- Appreciation of physicians and nurses who have good communication skills with patients and nurses and appreciation of them by the hospital ethics committee and announcement introduction of them as ethical mentors
5	Deficit knowledge of nurses about ethical issues and how to deal with them	Increasing the knowledge and skills of nurses about moral distress, consequences, and ethical decision-making solutions in situations that cause moral distress	- Holding a virtual educational session on the platform of Adobe Connect software, about the concept of moral distress, symptoms, and complications
			- Holding a face-to-face educational session on identifying and decision-making in situations that cause moral distress, focusing on examining moral cases
6	Observation of ethical dilemmas by nurses		- Question and answer sessions and presentation of cases and examples about moral distress in the virtual group about making decisions about situations that cause moral distress
7	The deficit in self-awareness and understanding of moral values	Empowering nurses to recognize the ethical issues and challenges of the nursing profession and solutions to reduce moral distress in nurses	- Holding a virtual educational session in the framework of the Adobe Connect program on ethical issues and codes and how to make ethical decisions
			- Conducting a face-to-face training session on recognizing ethical issues and how to make ethical decisions based on case studies and examples
			- Conducting question and answer sessions and presenting cases and examples in the virtual group, regarding the diagnosis of ethical issues and how to make ethical decisions in nurses
8	Aggressive behavior with patients	Improving anger control skills in nurses to reduce moral distress in nurses	- Holding a face-to-face training session on anger management skills
			- Presentation of training videos in a virtual group, about anger management skills
			- Holding question and answer sessions and presenting cases and examples in the virtual group, about anger control skills in nurses
			- Playing relaxing music in treatment departments for patients and nurses for two hours a day, every other day for a month

#### Stage 4) Identifying resources

In this stage of the model, the resources needed in the implementation of the interventions were identified and predicted by the researcher. The sources included professional expertise, audience, and people influencing the audience, existing policies, facilities, and equipment.

#### Stage 5) Planning evaluation methods

The quantitative evaluation to measure and determine the effects of the PRMD on the level of moral distress of nurses and the qualitative evaluation method, to examine and describe the effects of PRMD on participants; were conducted.

#### Stage 6) Setting an action plan

In this stage, the anticipated activities required to achieve specific goals and the time to achieve them have been determined.

#### Stage 7) Implementation of the plan

After designing the program based on Ewles and Sminett model, the program was implemented.

#### Post- implementation stage

##### Quantitative phase

The revised Hamric Moral Distress Questionnaire was used [33] in this phase. This questionnaire consists of 21 items on a 5-point Lickert scale, both the intensity and frequency of moral distress were measured by the questionnaire items. In this questionnaire, the frequency of moral distress on a Likert scale of 0 to 4 (never = 0 and very much = 4) and the intensity of moral distress on a Likert scale of 0 to 4 (not at all = 0 and very much = 4) were examined.

The scoring of each item was measured by integrating the frequency of moral distress multiplied by its intensity in the range of 0–16. Finally, the total scoring of 21 items is between 0–336. A higher score indicates more moral distress. Hamric has reported the reliability of this tool in the nursing community with Cronbach's alpha of 0.98. The content validity of the instrument in this study was assessed by four content experts on moral distress. The inter-rater agreement in this study was reported as 88%. The construct validity of the questionnaire has also been examined through hypothesis testing in this study (*p* = 0.005, *r* = 0.22).The revised Hamric Moral Distress Questionnaire was psychometrically evaluated by Soleimani et al. [34] in Iran in 2016 on 310 nurses were recruited from all hospitals affiliated with the Qazvin University of Medical Sciences from February 2014 to April 2015. The face validity and content validity of the tool have been investigated. The Content Validity Ratio (CVR) in this study is 0.86 and the Content Validity Index (CVI) is greater than 0.79. To determine the construct validity of this questionnaire, exploratory factor analysis with the Principle Component Method (PCA) was used to discover the internal correlation of the questions. Also, Kaiser-Meier-Elkin (KMO) sampling and Bartlett's Sphericity test (BT) were used to check sampling adequacy and sufficient sample size. Construct validity has been shown by 4 factors with an eigenvalue greater than one. The reliability of the questionnaire was also reported by calculating the internal correlation with Cronbach's alpha of 0.7.

Data analysis was done by using SPSS v.17. Descriptive statistics were used to describe the participants’ demographic personal and workplace characteristics and moral distress. Using a significance level of 0.05, parametric statistics (Repeated Measure ANOVA) were used to examine the difference between means of moral distress at different times. Bonferroni's post hoc test was used to determine the two-by-two difference between the averages of frequency, intensity, and total score of moral distress 4 different times.

##### Secondary qualitative phase

A qualitative content analysis, Face-to-face semi-structured interviews were conducted with duration of the interviews was between 45 and 60 min and began with a general question “Can you describe your experiences of attending in the PRMD?” Subsequently, the interviewer asked more specific questions: “Did the PRMD help reduce moral distress?” and “How did this program help you reduce moral distress?” Moreover, follow-up questions were asked to obtain more details about the objective of the study. Like the first qualitative phase, Granheim and Lundman stages and Lincoln and Guba criteria were used to analyze the data of this stage.

### Ethical considerations

The ethics committee of Shiraz University of Medical Sciences has approved this study by the number IR.SUMS.REC.1399.1078 to observe ethical considerations, the researcher, in addition to introducing himself and explaining the research objectives to the participants, asked them to complete the informed consent form. Before starting the interview, they could record audio and take notes. An overview will be published, and we reassure them that they are free to leave the study at any research stage.

## Results

### Pre-implementation stage

#### First qualitative phase results

Twelve nurses, including six female and six male nurses, were interviewed in this study. Participant characteristics are provided in Table 3. Due to analyzing data, seven categories highlighting the factors causing moral distress as experienced by nurses. The development process of one of the categories, showed in Table 4. categories and sub-categories were presented in Table 5.

**Table 3:** Demographic characteristics of participants (First qualitative study)

Demographic characteristics		Count	%
Age (years)	20–29	5	41.66
	30–39	5	41.66
	40–49	2	16.66
Gender	Male	6	50
	Female	6	50
Marriage status	Married	7	58.33
	Single	5	41.66
Educational degree	BSC	10	83.33
	MSc	2	16.66
Time employed (years)	1-5	5	41.66
	6-10	2	16.66
	11-15	3	25
	16-20	1	8.33
	21-25	1	8.33
Total	12

**Table 4:** Development process of one of the categories

Main theme	Category	Sub-category	Meaning units
Causes of moral distress	Lack of professional competence	The carelessness of colleagues in the care	- Failure to give serum or medicine to the patient by colleagues - Failure to care for patients by my colleague - Inadequate talk and attention of doctors to patients

**Table 5:** Factors causing moral distress in nurses (categories and sub-categories)

Category	Sub-category
Deficiency in professional competency	Negligence displayed by peers
	ineffective interdisciplinary participation
	Inadequate theoretical and practical knowledge
Unsuitable organizational culture	culture of professional domination of physicians
	insufficiency of nurse authority
Personal factors	nurses' lack of knowledge ethical issues
	inadequate sense of professional responsibility
Environmental and organizational factors	high workload
	insufficiency of personnel resources
	Facility and space constraints
Management factors	unsuitable division of tasks
	Insufficient supervision and regulation
Insufficiencies in proficient and efficient communication	manifestation of aggressive behavior whit patients
	Inappropriate interpersonal interactions
Nurses' observation of moral dilemma	observing treatment discrimination for homeless patients
	observing colleagues’ mistakes

##### Deficiency in professional competency

This category pointed to inability of nurses to perform their duties effectively and efficiently due to insufficient knowledge, skills, or experience that can lead to suboptimal patient care, increased risk of medical errors, moral dilemma and moral distress. This category consisted three sub-categories: “Negligence displayed by peers”, ineffective interdisciplinary participation” and “Inadequate theoretical and practical knowledge”

##### Unsuitable organizational culture

In this category, the nurses pointed to improper organization culture such as “culture of professional domination of physicians” and “insufficiency of nurse authority” that hinder open communication, limit the sharing of knowledge and expertise, and restrict the full utilization of nurses' skills and competencies and create moral distress.

##### Personal factors

This category related to individual and personal characteristics of nurses such as “nurses' lack of knowledge ethical issues” and “inadequate sense of professional responsibility” that can lead to failure to recognizing and addressing ethical issues in their practice, compromise patient care and caused moral distress.

##### Environmental and organizational factors

The nurses stated that “high workload”, “insufficiency of personnel resources” and “facility and space constraints” can affect the delivery of care, lead to burnout, decreased job satisfaction, and compromised patient safety and can also lead to moral distress among nurses.

##### Management factors

In this category the nurses reported “unsuitable division of tasks” and “insufficient supervision and regulation” can lead to increased workload, errors, adverse events and eventually caused moral distress.

##### Insufficiencies in proficient and efficient communication

The nurses stated “manifestation of aggressive behavior whit patients” and “inappropriate interpersonal interactions” can impact the quality of care provided by healthcare professionals. These factors can lead to burnout, decreased job satisfaction, compromised patient safety and caused moral distress.

##### Nurses' observation of moral dilemma

The nurses reported “observing treatment discrimination for homeless patients” and “observing colleagues’ mistakes” experience moral distress, as they may feel that they are unable to provide the care that their patients need and deserve. This can lead to feelings of frustration, guilt, and powerlessness, which can have negative implications for patient care and provider well-being and feeling of moral distress.

### Post-implementation stage

#### Quantitative study results

A virtual group was formed in the WhatsApp program and all participants members were added to virtual group. Then they were asked to complete the moral distress pre-test questionnaire, whose electronic link was made in google form and sent to the virtual group. The PRMD was implemented from the beginning of November 2021 and lasted for 3 months. Forty nurses including 21 female and 19 male, participated in this study. Participant characteristics are provided in Table 6.

**Table 6:** Demographic characteristics of participants (quantitative study)

**Demographic characteristics**		**Count**	%
Age (years)	20–29	9	22/5
	30–39	23	57/5
	40–49	8	20
Gender	Male	19	47/5
	Female	21	52/5
Marriage status	Married	31	77/5
	Single	9	22/5
Job Status	Nurse	33	82/5
	Head nurse	4	10
	Supervisor	3	7/5
Educational degree	BSC	35	87/5
	MSC	4	10
	PhD	1	2/5
Time employed (years)	1–5	9	22/5
	6–10	7	17/5
	11–15	15	37/5
	16–20	6	15
	21–25	2	5
	26–30	1	2/5
Employment Status	Permanent	26	65
	Contractual	6	15
	Training force	8	20
Total	40		

The post-test moral distress questionnaires were completed by the participants; immediately, one and two months after the intervention to check the effects of PRMD over time. Then the data were analyzed by SPSS software version 23.

Based on the statistical analysis of variance analysis of repeated measures, there was a significant difference between the average score of moral distress in nurses before the implementation of PRMD, immediately after the implementation of the program, one and two months after the implementation of PRMD, and the frequency score, intensity and total score of moral distress It has decreased after the implementation of the program. The results were reported in Table 7.

**Table 7:** Comparing the mean scores of moral distress in pre and post-implementation stages

	Before the implementation of the program	immediately after the implementation of the program	one month after the implementation of the program	two months after the implementation of the program	*P*-Value	F
	Mean ± SD	Mean ± SD	Mean ± SD	Mean ± SD		
Frequency of moral distress score	2/390 ± /221	2/297 ± /226	2/207 ± /270	2/122 ± /288	000/0	22/098
The intensity of moral distress score	2/283 ± /193	2/212 ± /182	1/925 ± /222	1/775 ± /248	000/0	39/296
Moral distress total score	139/750 ± 19/516	129/575 ± 18/620	106/300 ± 19/602	92/750 ± 20/124	000/0	75/347

#### Secondary qualitative study

Six nurses including 3 female and 3 male participated in this study. Qualitative analysis of 6 interviewed nurses resulted in two major themes highlighting the consequences of PRMD on them. The themes were as follows: (1) moral empowerment of nurses (2) Promotion of ethical climate. Themes and subthemes were presented in Table 8.

**Table 8:** Themes and Subthemes in the Post-Implementation stage

Theme	Subtheme
moral empowerment of nurses	Improving moral knowledge and skills
	Improving professional communication
	Promotion of teamwork
Promotion of ethical climate	Modifying apportion duties methods
	Workload reduction
	Involvement of managers in improving the ethical climate

### Moral empowerment of nurses

Participants in the program state that the program, morally empowered them. This empowerment has happened through Improving moral knowledge and skills, Improving professional communication and promotion of teamwork.

Participant number 2 has stated in this regard:

"I didn't know much about the ethical principles that a nurse should observe, and I didn't know what to do when I feel that something unethical is happening, but by participating in this program and the educational issues and scenarios that were executed and talking about them in the group colleagues, helped me a lot to empower me in dealing with nursing ethics issues”.

Also, participant number 4 stated:

"Participating in this program was a very enjoyable experience for me because no one had ever taught moral issues and how to deal with them in the form of scenarios, clips, and discussions. On the other hand, I realized that teamwork and thinking with colleagues can play a very good role in managing nursing ethical issues and reducing moral distress."

### Promotion of ethical climate

The participants also stated that this program has improved the moral environment through modifying apportion duties methods, workload reduction and involvement of managers in improving the ethical climate.

In this regard, participant number 3 stated:

"For years, there was a problem of apportion duties methods in our ward, but no one was doing anything to solve it. The implementation of this program and the raising of this problem in the group of managers and the common thinking of managers and nurses caused this problem to be solved and also the workload Reduce."

Also, participant number 5 said in this regard:

"One of the benefits of implementing this program was that it made managers ask nurses for their opinions to make changes and involve nurses in decision-making, which had a great impact on improving the moral atmosphere of the work environment."

### Program evaluation stage

Integration of quantitative and qualitative data to evaluate and refine outcomes of programs related to nurses, patients, and healthcare system was done. The results and effects of the program were consistent with both quantitative and qualitative approaches and the results were mutually supportive. The findings of the quantitative stage showed a decrease in the overall mean score, severity and frequency of moral distress in nurses. These findings were consistent with the themes of revealing moral capabilities in nurses and improving the moral environment that was obtained in the secondary qualitative section and indicated the effect of the intervention.

## Discussion

Using mixed methods and a pre-post design, were evaluated the effectiveness of PRMD on nurses, patients, and the health care system.

The quantitative results showed that the level of moral distress in participants decreased immediately after the implementation of the program and also over time, which indicates the positive effect of PRMD on them. The PRMD program was a combination of educational, managerial, and, environmental interventions to reduce moral distress. Educational interventions in this program aimed at acquiring knowledge and skills about moral distress, causes and consequences of MD, codes of ethics in nursing, communication skills, and anger control skills. Also, by presenting ethical cases and practicing practical scenarios in the face-to-face classes, strengthened the problem-solving skill, self-expression, and reflective debriefing, and nurses' learning was expanded and deepened.

In the studies that were done in Iran reported interventions such as educational workshops, moral empowerment programs, social work interventions, nursing ethics huddles, and multifaceted resiliency bundle interventions for managing and reducing moral distress [3, 35–37].

Ethics training gives nurses the necessary tools to make decisions and develop personal coping skills. Moral education may also improve self-confidence, reduce fear, and improve the ability to deal with complex moral dilemmas [31]. Many researchers have emphasized the importance of creating morally challenging situations, redefining, and explaining moral distress. [13, 32–34] The studies conducted have also positively evaluated the effect of educational interventions on reducing moral distress [12, 35–37]. The training interventions were accompanied by Reflective Debriefing' facilitated discussions, which made the participants involved with the provided training and practice and repeat what they learned [38]. In our study; one Nurse Ethicist facilitated a group discussion of a patient case that includes reflection, clarification of stakeholder values & ethics education. This method has also been examined in studies and its positive effects have been reported [39–41]. In Iran; Ghahremani et al. reported professional ethics workshop with virtual follow-up reduced the intensity and level of moral distress and follow-up through social network reduced the frequency, intensity and the level of moral distress among nurses [36]. Also Sadeghi-Gandomani et al. emphasized on implementing empowerment and educational programs to minimize the severity and frequency of moral distress among iranian nurses [42].

Managers' lack of support for nurses and nurses' lack of participation in decision-making are among the factors that play an important role in creating moral distress in nurses, which is confirmed by the results of our study and many studies [38, 43, 44]. The management interventions used in this program were focused on the gaps identified in the initial qualitative phase. In this program, managers actively participated in training classes and designed management solutions with nurses. Also, managers participated in intra-ward meetings with nurses and identified processes that increase workload, and reviewed and corrected apportion duties methods in the wards. By participating in the formulation of ethical clinical policies and activities and with proper support (including the opportunity for self-reflection and the opportunity to cooperate properly with other colleagues and other members of the treatment team in management), nurses create an environment that can provide safe, competent and ethical care [45].

In Iran, nursing ethics and moral stress have been the focus of several studies highlighting the importance of addressing these issues in healthcare management. These studies provide valuable insights into the unique challenges faced by Iranian medical personnel and possible measures that could be implemented to boost morale and reduce moral distress.

Abbasi et al. recommended that nursing managers and hospital managers participate in moral empowerment programs [38]. Atashzadeh-Shoorideh et al. found that moral leadership plays an important role in reducing moral stress in nurses and highlighted the importance of active involvement of managers in ethical decision-making processes and support for nursing staff in dealing with ethical issues. In a study by Rushton et al., the authors emphasize the importance of ethical leadership in healthcare institutions, especially in terms of fostering a culture of ethical practice and solving moral problems among nurses [46]. Also, Lamiani et al., have confirmed the importance for healthcare managers to invest in "intangible goods," such as a positive ethical climate, team collaboration, and support to prevent moral distress, and increased job satisfaction [47]. In conclusion, the managerial interventions used in this program support the findings of studies that emphasize the importance of considering nursing ethics education and moral distress. By incorporating these principles into the program, it can significantly improve the ethical climate for nursing staff and, in turn, improve the quality of care provided to patients.

In this study, environmental interventions were designed to make changes in the environment in which ethical care takes place. Identifying and appreciating physicians and nurses who have appropriate communication skills with patients and colleagues, playing relaxing music in treatment wards for patients and nurses to create relaxation in staff and patients and reduce aggression, introducing moral mentors in the hospital, and strengthening collaboration between Professionals were among the environmental interventions to improve the ethical climate. Also, the qualitative findings after the PRMD; confirmed the effectiveness of environmental interventions in improving the ethical climate. The participants confirmed that this program was able to provide the conditions for performing moral actions through modifying apportion duties methods, workload reduction, and involvement of managers in improving the ethical climate. Creating an ethical climate in the hospital and emphasizing the psychological empowerment of nurses and increasing their knowledge of moral distress and complications and ways to reduce it has been emphasized in many studies [48, 49]. Multiple qualitative and quantitative studies in the nursing literature have found that a positive ethical climate facilitated less moral distress amongst nurses [50–52]. In a study conducted by Abbasi et al., through the implementation of the empowerment program and conducting interventions such as training on moral distress and its complications, training skills to deal with moral distress such as problem-solving skills, self-expression, presenting moral scenarios, And discussions about them and teaching communication skills to nurses have been able to reduce their moral distress [38].

After the implementation of PRMD, the participants in qualitative interviews reported increasing their moral knowledge and skills, improving professional communication, and Promotion of teamwork and moral empowerment was achieved. Moral empowerment of nurses is provided through training and promoting the level of psychological knowledge, effective communication skills, and changing the view of the health team toward each other [53].

## Conclusion

Moral distress is a multi-dimensional and complex phenomenon that multi-dimensional and multi-disciplinary interventions should be designed to reduce. The result of our study showed that interventions based on needs and culture and facilities and conditions of healthcare systems can reduce moral distress in nurses. The use of different educational tools such as lectures, PowerPoint, educational clips, and different teaching methods such as face-to-face training, virtual training, role-playing, and face-to-face scenario exercises can increase the depth of the taught content and have a greater effect on reducing moral distress. Also, the participation of nurses and managers in designing solutions and strategies has a very effective role in the effectiveness of interventions.

Our study faced many limitations due to the covid-19 pandemic, and the number of participants was also limited. Therefore, it is suggested to implement this program in more medical centers and a larger number of nurses.

## Data Availability

The datasets used and analyzed during the current study are available from the corresponding author on reasonable request.
